# Identification of Protein Interaction Partners in Mammalian Cells Using SILAC-immunoprecipitation Quantitative Proteomics

**DOI:** 10.3791/51656

**Published:** 2014-07-06

**Authors:** Edward Emmott, Ian Goodfellow

**Affiliations:** ^1^Division of Virology, Department of Pathology, University of Cambridge

**Keywords:** Biochemistry, Issue 89, mass spectrometry, tissue culture techniques, isotope labeling, SILAC, Stable Isotope Labeling of Amino Acids in Cell Culture, proteomics, Interactomics, immunoprecipitation, pulldown, eIF4A, GFP, nanotrap, orbitrap

## Abstract

Quantitative proteomics combined with immuno-affinity purification, SILAC immunoprecipitation, represent a powerful means for the discovery of novel protein:protein interactions. By allowing the accurate relative quantification of protein abundance in both control and test samples, true interactions may be easily distinguished from experimental contaminants. Low affinity interactions can be preserved through the use of less-stringent buffer conditions and remain readily identifiable. This protocol discusses the labeling of tissue culture cells with stable isotope labeled amino acids, transfection and immunoprecipitation of an affinity tagged protein of interest, followed by the preparation for submission to a mass spectrometry facility. This protocol then discusses how to analyze and interpret the data returned from the mass spectrometer in order to identify cellular partners interacting with a protein of interest. As an example this technique is applied to identify proteins binding to the eukaryotic translation initiation factors: eIF4AI and eIF4AII.

**Figure:**
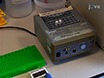


## Introduction

An essential step in understanding protein function is identification of relevant interacting proteins. Where such proteins are unknown there are a number of techniques available, each with their own merits and drawbacks. These include the yeast two-hybrid system, pulldown assays using recombinant protein, as well as tandem affinity purification or TAP-tagging^1^^,^^2^.

A more recent addition to these techniques is the combination of affinity purification of a protein of interest from a relevant mammalian cell line, followed by quantitative mass spectrometry using stable isotope labeling of amino acids in cell culture (SILAC)^3^. This has advantages over the yeast two-hybrid approach in that cell localization and post-translational modifications are not perturbed, as well as advantages over traditional TAP-tagging in that it is a quantitative rather than qualitative approach allowing the user to readily distinguish non-specifically interacting proteins and contaminants, from host factors that bind specifically. Further, as a sample is typically analyzed whole, rather than as individual protein bands, proteins of interest are not masked by similarly migrating proteins on a gel, nor do they typically need to be present at sufficient levels to be visible after staining, leading to increased numbers of confidently identified proteins ^4^.

To demonstrate this technique, GFP fusions of the closely related eukaryotic translation initiation factor eIF4AI and eIF4AII that share over 90% amino acid identity were investigated by SILAC-immunoprecipitation quantitative proteomics. Human eIF4AI and II were cloned into pEGFP-C1 to form a fusion protein where GFP is fused to the N-terminus of eIF4A. To avoid the need for generating stable cell lines transient transfection was used to deliver these constructs to stable isotope labeled 293T cells.

Cells were first labeled for two weeks in SILAC cell culture media, followed by transfection of plasmid DNA encoding a protein of interest. Cells were then lysed, the protein concentration normalized, and equal amounts of lysate affinity purified on anti-GFP agarose. Equal amounts of eluate were then combined and submitted for LC-MS/MS analysis. The results of this analysis are then processed to identify high confidence protein:protein interactions (**Figure 1**).

SILAC immunoprecipitation enables the identification of not only direct interactions but also low affinity or indirect interactions with protein complexes^4^. Using this system, eIF4AI and II immunoprecipitations allowed reproducible and confident identification of the primary binding partner eIF4G (isoforms I/II and III)^5^, as well as indirect interactions with eIF4E, and numerous components of the eIF3 complex.

## Protocol

### 1. Generation and Passaging of SILAC-labeled Cell Lines

Note: the use of Trypsin-EDTA must be avoided at all stages of passaging and preparation of experimental samples for analysis as the trypsin may contain unlabeled amino acids, which would lead to incomplete labeling of samples.

1. To prepare a bottle of SILAC media add a 0.5 ml aliquot of an appropriately SILAC-labeled arginine (84 mg/ml in PBS) and lysine (146 mg/ml in PBS) to a 500 ml bottle of Arg/Lys free DMEM (containing L-glutamine).
2. Next, add 50 ml of dialyzed FBS and 5 ml of penicillin/streptomycin to the 500 ml bottle. Note: HEK 293T cells (ATCC) should be maintained in DMEM media lacking arginine and lysine and supplemented with light (R0K0), medium (R6K4) or heavy (R10K8) amino acids, dialyzed fetal bovine serum (10 kDa cutoff), and penicillin/streptomycin. Cells must be maintained in media for a minimum of 5 cell divisions to ensure complete labeling. In most cases cells are readily labeled in ≥2 weeks.
3. To split cells for passaging, cells should be dislodged from the monolayer by hitting the flask. Alternatives include the use of cell scrapers or by using enzyme-free, PBS-based cell-dissociation buffer. Note: To conserve media, cells should be passaged in T25 flasks and larger cell numbers generated only immediately prior to an experiment.
4. 24 hours prior to transfecting cells, seed 3.5 x 10^6^ SILAC-labeled cells into a single 10 cm^2^ dish for each experimental condition under investigation.

### 2. Transfection of Labeled Cells with pEGFP-fusion Constructs

1. Remove the media from the cells and replace it with 9 ml of antibiotic-free SILAC DMEM (Light, Medium or Heavy) media. Note: Antibiotics should not be added to the media since they may interfere with the efficiency of liposome-based transfection reagents.
2. Prepare a mix of 10 μg of the appropriate plasmid (epEGFP-C1, pEGFP-eIF4A-I, pEGFP-eIF4A-II) in 500 μl of antibiotic-free SILAC DMEM and mix it with 500 μl of antibiotic-free SILAC DMEM containing 10 μl of transfection reagent (*e.g.*, lipofectamine 2000). Mix the reaction thoroughly by pipetting it up and down several times.
3. Incubate the reaction mix at room temperature for 20 min and then add drop-wise to the cell monolayer. Rock the plate gently from side to side.
4. Incubate the transfected cells at 37 °C and 10% CO_2_ for 24 hr. Note: If the expression of a protein of interest results in any apparent toxicity, then it may be necessary to reduce the amount of plasmid transfected and/or the duration of the subsequent expression period.

### 3. Harvesting Cell Lysates

1. Harvest cells from the dish into ice cold PBS using a cell scraper. Collect cells by centrifugation at 220 x g, 4 °C for 5 min. Wash the cells a further 3x in 10 ml of ice-cold PBS.
2. Resuspend the cell pellet in 200 μl of cell lysis buffer (10 mM TrisCl/pH 7.5, 150 mM NaCl, 0.5 mM EDTA, 0.5% NP40) containing freshly added protease inhibitor cocktail III at 1x concentration and RNase cocktail (optional) at 5 μl per ml. Note: For proteins with known nucleic acid binding activity, it may be necessary to add nucleases to the lysate prior to precipitation. In cases where nuclease is added, samples should be incubated on ice for 30 min on ice, with pipetting every 10 min. Extracting total nucleic acid from a small fraction of the sample and analysis by agarose gel electrophoresis can test the effectiveness of the nuclease.
3. Centrifuge samples at 13,000 x g, 4 °C for 10 min retain the supernatant as the soluble cell lysate.
4. The concentration of the cell lysate should be assessed by BCA assay according to the manufacturer’s instructions.
5. Use lysis buffer containing protease inhibitor cocktail III to normalize protein concentration in a final volume of 500 μl.
6. Adjust the volume to 1 ml with the addition of 500 μl of dilution buffer (10 mM TrisCl/pH 7.5, 150 mM NaCl, 0.5 mM EDTA) containing protease inhibitor cocktail III at final 1x concentration. A 50 μl aliquot of the sample should be retained as the sample input and the lysate kept on ice whilst preparing anti-GFP beads (*e.g.*, GFP-trap). Note: Typically yields vary between 1-3.5 mg of protein in the final 1 ml sample. Whilst the above buffers are suitable for many proteins of interest, for others it may be necessary to modify buffer components to ensure the bait protein is solubilized and to maintain protein-protein interactions. Possible alterations include the buffering agent (Phosphate, HEPES), salt concentration (150-500 mM), the choice of detergent, or other additives.

### 4. Binding to Anti-GFP Beads

1. Briefly vortex the bead slurry to resuspend the beads. Using a 200 μl pipette tip with the end cut off, transfer 25 μl of beads per sample to a fresh tube. Note: The user should prepare the beads for a single SILAC experiment as a mastermix to minimize sample-to-sample variation.
2. For each 25 μl of bead slurry, add 20 volumes (1,500 μl per 75 μl slurry) of dilution buffer, and centrifuge the beads at 2,700 x g for 5 min. Next, wash the beads a further 2x in 20 volumes of dilution buffer.
3. Add 100 μl dilution buffer per 25 μl bead slurry. Using a 200 μl tip with the end cut off, transfer 85 μl of this resuspended slurry to each of the SILAC-labeled samples from step 3.6.
4. Incubate the samples with beads on a rotator at 4 °C for 2 hr.

### 5. Washing, Elution, and Preparation of Samples for MS Analysis

1. Centrifuge samples at 2,700 x g, 4 °C for 5 min. 50 μl of the supernatant should be retained as the unbound sample, with the remainder of the supernatant discarded.
2. 1ml dilution buffer should be added to each tube to resuspend the beads and the sample centrifuged at 2,700 x g, 4 °C for 5 min. The supernatant should be discarded. This should be performed twice.
3. Elute protein from the beads by the addition of 50 μl of 2x SDS loading buffer, and heating at 95 °C for 10 min. Pellet the beads by centrifugation at 2,700 x g for 2 min at 4 °C
4. Retain the supernatant in prelubricated tubes, where it can be then stored at -80 °C until required. For submission to a mass spectrometry facility, mix labeled samples 1:1:1 (*e.g.*, 10 μl of each) and submit the mixed sample. Note: At this point samples may be tested by western blot, silver-staining or other means to test for interaction with known/unknown binding partners. An example is given in **Figure 2** showing specific binding of a known interaction partner – eIF4G by western blot, and the appearance of silver staining bands present in pulldown samples from a protein of interest, but not a control sample.
5. Submit sample for LC-MS/MS analysis. Note: once satisfied that a tagged protein of interest is successfully binding interaction partners, equal volumes of each labeled sample (light, medium and heavy) are combined and submitted for LC-MS/MS analysis. It is usual to submit 30 μl total of an IP sample for analysis. This would entail mixing 10 μl of Light-labeled sample with 10 μl of medium-labeled and 10 μl heavy-labeled samples to give a 30 μl total.

### 6. Data Analysis I: Understanding the Results, and Removal of Low-confidence Identifications

Note: A list of the column headings returned by the Proteome Discoverer software is given in **Table 1**. Different software (*e.g.*, MSQuant, MaxQuant) will return different headings, however, only a subset of these are required for analysis and these are common to the various software packages. Data should always include an accession number for each protein identified, ratio’s comparing each sample ratio (light vs medium, light vs heavy, medium vs heavy etc), the number of unique peptides identified, and some form of false positive rate or confidence indication.

1. Before going through the data, first copy the raw data to a new spreadsheet. From this spreadsheet remove all the columns except those giving the accession number, number of unique peptides, ratios comparing samples, ratio variability, and the protein description. If replica experiments were performed, these should be combined into a single Excel file, with each experiment appearing on a separate tab. Note: Low confidence data includes proteins identified by only a single unique peptide, and those where quantification was not possible.
2. Use Excels’ ‘sort’ function to order the data by the number of peptides and remove the entries for proteins lacking more than one peptide. Then sort by ratio and remove proteins that lack SILAC ratios (unquantified proteins).
3. Convert SILAC ratios to a log_2_ values using the formula: ‘=log(***SILAC Ratio***,2)’, where ‘SILAC Ratio’ is substituted for the cell identifier. Note: As a SILAC ratio results in any data for proteins showing a decrease in abundance being restricted to values between 0 and 1, it is usual to convert a SILAC ratio to a log_2_ SILAC ratio, as this means proteins both increased or decreased in a sample are represented on a log scale where 2 or -2 represents a 4-fold increase or decrease in abundance, and 3 or -3 a 9-fold increase or decrease in abundance respectively. Following this transformation, the data should fit a Gaussian distribution centered around a log_2_ SILAC ratio of 0. An example of this is given in **Figure 3**.
4. Create new columns in the excel file and calculate log_2_ SILAC ratios for all the sample/mock columns. For conversion of a mock/sample ratio to a sample/mock ratio, use the formula: “=1/ratio”

### 7. Data Analysis II: Selecting High Confidence Interactions for Further Study

1. Open Graphpad Prism, select New>Data Table and Graph ... Select ‘column’ from the list on the left hand side of the window, and select the Enter/Import data>Enter replica values, stacked into columns options. Press ‘Create’.
2. Select and copy a given log_2_ Sample/Mock SILAC ratio column from the Excel spreadsheet into the new Prism file.
3. Click the dropdown ‘Insert’ menu and select ‘New analysis’. Under column analysis select ‘Frequency distribution’. Keeping the default options click ‘OK’. Note: This step generates a histogram illustrating the number of proteins identified at a given ratio. This should form a Gaussian distribution.
4. In the ‘Results’ folder a new ‘Histogram’ section will have been generated. Select the frequency distribution section.
5. Click the dropdown ‘Insert’ menu and select New analysis>XY>Nonlinear regression (curve fit). Click on ‘Gaussian distribution’ and click OK. Note: This step fits a curve to the frequency distribution data generated in step 7.3, yielding values that can subsequently be used to calculate thresholds for significance.
6. In the results window that appears, the mean and standard deviation are given. Generate a threshold by adding 1.96 standard deviations to the mean. Note: These values represent the mean and standard deviations of the Gaussian distribution, not the total dataset. 1.96 standard deviations would place a threshold at the 95% confidence limit (p≤0.05). 2.58 SD would give 99%, and 3.3 SD 99.9%. The threshold should be determined individually for each replica experiment as it may vary.

### 8. Data Analysis III: Merging Replica Datasets

Note: To be confident in identifying interaction partners a typical SILAC pulldown experiment should ideally be performed three times, with the medium and heavy labeled samples switched for one of the repeats to control for any effect of the media on the results. A protein that shows up as interacting in at least two of the three experiments, with the ‘switched’-media sample ideally representing one of these, is a high confidence interaction.

1. Return to the Excel file, create a new tab called ‘Combined’. Label column A ‘Accession’ and copy all the accession numbers from each of the individual experiments into this single column.
2. Select the ‘Data’ tab, and then the ‘Remove Duplicates’ option.
3. Create columns for the SILAC ratios, and ratio variability from each experiment as well as for the protein name/description column.
4. In the description tab, use the vlookup formula to collect the description of each accession number. Drag the formula down the column to fill in the protein description. The vlookup formula is: “=vlookup(***AccessionNo***, ***WheretoLook***, ***ColumnsAccross***, False)”, where: ***AccessionNo*** is $aX with X being the row number. ***WheretoLook*** is the ‘Experiment tab Name’!$a$2:$Y$Z where Y and Z are the bottom right piece of data on the spreadsheet being referenced. ***ColumnsAccross*** is the number of columns across from the accession number in column A a desired value lies. Note: For example, if the Accession number is in column A, and the data of interest lies in column E. The value here would be 5 (column A counts as 1). As accession numbers were pooled in step b, it is most likely that vlookup will not obtain all the required names from one experiment. Where it returns N/A, modify the formula to obtain the description from each experiment in turn until all the descriptions are acquired.
5. To highlight interacting proteins in the combined dataset, select each ratio column individually and click on the Home tab, followed by Conditional Formatting>Highlight cells rules>More rules.
6. Select style ‘Classic’. Format only cells that contain ‘Cell value’, ‘Greater than or Equal to’. In the box, type in the 1.96 standard deviation value, and click ‘OK’.
7. Assess the ratio variability for each positive interaction. If subtracting the % variability would drop a ‘hit’ below the threshold value, this should be treated with caution. Note: This represents how consistent the individual ratios for each peptide that together make up a proteins’ SILAC ratio are, and is given as a percentage. When the ratio variability is taken into account and a protein gives a SILAC ratio above the threshold, this protein represents a hit. If the ratio variability gives a range that falls below the threshold, the ‘hit’ may represent a contaminant.
8. Repeat this for each of the results columns and compare across experiments. Highlighted proteins identified in two or more experiments represent a high confidence interaction. Note: Depending on the database used to assign protein accession numbers, in different experiments a protein may have been identified under a different, or even multiple accession numbers. It is important to check this to ensure that proteins are not accidentally omitted.

## Representative Results

In a typical SILAC pulldown experiment, the vast majority of identified proteins (>90%) represent contaminants as well as proteins binding non-specifically to the affinity matrix and this is illustrated in **Figure 2B**, even when washing protocols remove a majority of cytoplasmic contaminants such as GAPDH (**Figure 2A**). However, the clustering of non-specifically binding proteins in a normal distribution allows proteins that specifically bind to a protein of interest to be distinguished as these have higher sample/mock ratios than the background. Whilst contaminants should theoretically cluster around a log_2_ SILAC ratio of 0, this is not necessarily the case, an example of this is given in **Figure 3**. Possible reasons for this include imperfect SILAC labeling of cells, loading unequal volumes or concentrations of lysate onto the anti-GFP beads, accidental loss of beads during purification or unequal mixing of samples at the end of the purification procedure^3^. However, assuming data are analyzed based on a threshold standard deviation from the mean of the normally distributed contaminants, minor shifts in the centering of the data should not affect the quality of the results.

When comparing differences in protein interactions between two related proteins, a similar situation may occur where one protein of interest is produced to higher levels within cells than a second (either due to variation in transfection efficiency, or an intrinsic property of the protein or mRNA). Some variation in expression (*e.g.*, **Figure 2A**) can be corrected for by analyzing the SILAC ratio for these two samples. In this example these would be the GFP-eIF4AI and GFP-eIF4AII samples. By analyzing the 4AI/4AII SILAC ratio as discussed in section 7, it is possible to identify proteins whose binding varies significantly between isoforms.

In **Figure 4**, a representation of the eIF4A-binding proteins identified in one of the replica experiments conducted is shown illustrating the coverage of the initiation factor complex this protocol achieved. The highest ratios were typically observed with eIF4G, which binds directly to eIF4A, with lower ratios for eIF4E, which binds to eIF4G at a site away from the eIF4A-binding site. Lower ratios were observed for members of the eIF3 complex. However, this clearly illustrates that the experiment satisfactorily identified both direct and indirect binding partners of eIF4AI and II. As might be expected from their high sequence identity^6^, protein:protein interactions appeared largely conserved between the two isoforms in this experimental system^7^^,^^8^. A selection of some of the interacting proteins are given in **Table 2**, illustrating the data format.

**Table:** 

**Column Heading**	**Description**
**Accession**	**Displays the accession number for the sequence**
Coverage	Proportion of the protein sequence covered by the identified peptides
♯PSM	Peptide spectral match
**♯peptides**	**Total number of unique peptides identified for a protein**
♯AAs	The length of a protein in amino acids
MW (Da)	The molecular weight of a protein in Daltons. Excludes modifications
calc. PI	The theoretical isoelectric point of a protein
Score	The total score of a protein (which represents the sum of the individual peptide scores). The exact score required for significance will vary between experiments. A MS facility will usually apply a 5% false discovery rate cutoff.
Sequence	The sequence of amino acids constituting the protein
**Ratio**	**The relative intensity of peptides in a named labeled sample, compared to a second labeled sample**
Ratio Count	The number of peptide ratios that were used to calculate the a given protein ratio
**Ratio variability (%)**	**The variability of the individual peptide ratios used to calculate a given protein ratio**
**Description**	**The name of the protein**

**Table 1. Standard column headings from a Proteome Discoverer report.** Whilst useful information can be gained from all of these columns, those critical for this analysis are shown in bold.

**Table:** 

**Accession**	**Peptides**	**4AI/Mock**	**4AII/Mock**	**Name**	**SILAC analysis**
A8K7F6	21	100	1	eIF4AI	‘Bait’ protein
Q14240	22	0.01	90.855	eIF4AII
G5E9S1	25	47.575	30.53	eIF4GI	Interacting proteins
Q59GJ0	5	11.778	10.619	eIF4GII
P06730	3	7.22	7.57	eIF4E
Q5T6W5	4	0.685	0.646	hnRNPK	Non-specifically binding contaminants
P62805	1	0.531	0.498	Histone H4
H6VRG2	18	-	-	Keratin-1	Environmental contaminants
P35527	11	0.01	0.01	Keratin-1 cytoskeletal 9

**Table 2. Typical Data from a SILAC immunoprecipitation experiment.** Giving example data for a protein of interest/bait (high peptides, high ratio), proteins interacting with a protein of interest (high/low peptides, high ratio), non-specifically binding proteins (high/low peptides, ratio falls below cutoff – in this experiment 0.96), and environmental contaminants (often high peptides, negative ratio/below threshold).


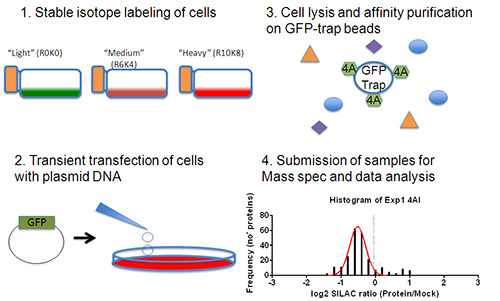
**Figure 1. Experimental Plan. **Firstly cells are grown in media lacking Arginine and Lysine and substituted with stable isotope labeled Arginine and Lysine for 2 weeks (**1**). (**2**) Cells are seeded into 10 cm^2^ dishes and transiently transfected with plasmids encoding GFP (Mock) or GFP fusion proteins (Samples). (**3**) Cells are lysed and GFP or GFP fusion proteins are immunoprecipitated from cell lysates. (**4**) Samples are combined in a 1:1 ratio and submitted for LC-MS/MS analysis. Data is then analyzed to remove low confidence protein identifications and to select a level of protein enrichment corresponding to genuine interacting proteins.


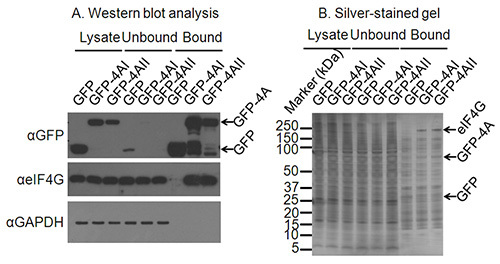
**Figure 2. Confirming suitable immunoprecipitation conditions. A**) Western blot analysis of cell lysates, as well as the unbound and bound fractions from the immunoprecipitation confirms expression and immunoprecipitation of the protein of interest. A western blot against GAPDH confirms depletion of non-interacting proteins and a further western blot a known interacting partner of eIF4A confirms the successful immunoprecipitation of proteins binding to the protein of interest. **B**) Where interacting partners of a protein of interest are not known, a silver-stained gel may confirm immunoprecipitation of interacting proteins. On this silver-stained gel bands for GFP and GFP-eIF4AI/II are clear, and a band migrating at the correct size for eIF4G is present only in the GFP-4AI/II-bound lanes and not in the GFP control lane.


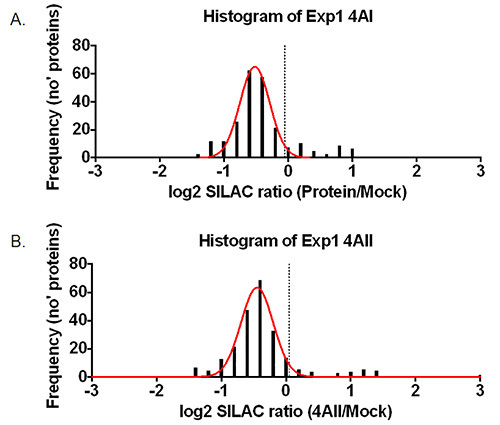
**Figure 3. Representative results. **Histogram showing the distribution of protein ratios from one repeat of (**A**) GFP-eIF4AI or (**B**) GFP-eIF4AII pulldown. The 1.96 standard deviation cutoff is marked with a dashed line. Interacting proteins falling outside the normally-distributed contaminants are evident from ~0.25 and 1 in (**A**), and ~0.3-1.5 in (**B**).


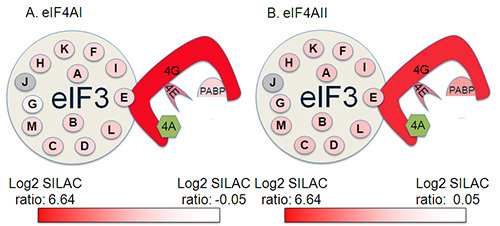
**Figure 4. Identification of eIF complex members from a single replica of a SILAC IP experiment. **Proteins in green were the protein of interest used for the pulldown Interacting proteins are shaded from red to white according to log_2_ SILAC ratio in the SILAC IP with red being the most abundant protein in the analysis, and white being the 1.96 SD cutoff. Proteins shaded in grey were not identified in this analysis.

## Discussion

The SILAC pulldown strategy described here represents a very sensitive and powerful means of detecting novel protein:protein interactions, and furthermore allows the rapid and simple discrimination of altered binding patterns between closely related samples of interest. In this example this technique was used to investigate the protein:protein interactions of the eIF4AI and eIF4AII proteins^6^. To the author’s knowledge, this is the first study in the literature exploiting the utility of SILAC proteomics to investigate the cellular interactome of these two isoforms of eIF4A.

The approach as described above uses a GFP-tag and anti-GFP beads^9^^,^^10^ and therefore modifications may be required to enable this approach to be used for a specific protein of interest, for example whether the tag is placed at the N- or C-terminus of a protein. Where possible, western blots or functional assays should be performed to detect binding of a known protein interaction partner. Should a protein not tolerate fusion with a GFP tag, other tagging or pulldown strategies have been applied to SILAC pulldowns using both other tags (FLAG^11^, Biotin^12^, STREP (own data, unpublished)), or by using primary antibodies against a protein of interest where siRNA knockdown of the target protein provides a control sample^13^. Such experiments have been described elsewhere in the literature, but in brief, step 1 and steps 5.4-8 would be applied as above, with steps 2-5.3 modified as appropriate for the expression/pulldown system of choice using equal protein inputs as described in steps 2.4-2.5. As the quantification stages allow non-specific binding proteins to be removed at the analysis level, it is recommended to omit pre-incubation with control beads, or high salt washes in order to preserve low-affinity protein:protein interactions with a protein of interest. A nuclease may be included or omitted from this protocol according to the specifics of a particular experiment. For example: as the proteins used in this method are RNA helicases, RNase cocktail was included in the protocol to remove indirect interactions mediated via the RNA (step 3.2). In some cases however, there could be benefit from running parallel experiments with and without nuclease to identify nucleic acid dependent interactions.

Within this protocol, the switching of ‘medium’ and ‘heavy’ samples in replicate experiments is recommended to control for variation introduced by differences in the SILAC media or cell growth. An alternative control involves the sequential switching of all three (‘light’, ‘medium’, and ‘heavy’) media in replica experiments. Whilst this approach is potentially more stringent, it does increase the complexity of the analysis, as in at least one replicate, a protein of interest will be produced in ‘light’ labeled cells and so it is necessary to distinguish between proteins consistently identified in ‘light’ samples (environmental contaminants such as keratins), and those that are only enriched in ‘light’ samples when bound to a protein of interest.

Whilst the use of quantification data allows discrimination of specific- from non-specific interactions through use of a threshold, inevitably some genuine interactions may be discarded. The approach above is a simple and rapid approach to identifying protein:protein interactions that can be easily attempted by researchers with no previous experience with mass spectrometry, or analysis of large protein:protein interaction datasets. For most uses this is more than sufficient for identifying novel proteins of interest. Further modifications to this approach to help reduce this data loss are described elsewhere in the literature and include the use of a protein frequency library where known contaminant proteins for a specific set of experimental parameters (cell line, bead matrix, buffer conditions) may be excluded^10^^,^^14^. However, depending on the particular experimental parameters it may be necessary to run a number of control experiments to generate a bead proteome and this can therefore increase both the expense and complexity of the experiment. Further information on this technique is available from the www.peptracker.co.uk website^14^.

It should also be noted that the protocol described above involves mixing differently labeled samples at the end of the immunoprecipitation process (termed a Mixing After Purification – MAP SILAC experiment). This is done as protein:protein interactions occur at a given equilibrium^15^. It should be noted that other groups have combined this MAP SILAC approach described in this protocol with incubation of the samples prior to pulldown (Purification After Mixing – PAM SILAC) for different lengths of time (20 min to 2 hr have been used in the literature)^15^^,^^16^. Based on how rapidly a protein ratio drops towards 1:1, it is possible to qualitatively investigate binding affinities and to define proteins as stable or dynamic interacting proteins^15^.

In summary, SILAC pulldowns represent a very powerful means of identifying proteins interacting with a given protein of interest, in a physiologically relevant setting. The technique can be very easily adapted to a number of different purification strategies, allowing its application to any given protein of interest. Quantification of results vastly simplifies identification of genuine interactions, and permits relaxation of stringent buffer conditions used to remove non-specific binders, and thus preserves low affinity interactions. As up to three samples can be compared in the above strategy, the technique has clear strengths in comparing differences in protein binding between different protein isoforms, mutant proteins, or the effect of pharmacological inhibitors. As whole gel slices are analyzed rather than individual bands that stain by Coomassie, the numbers of proteins identified at high confidence are typically higher than those identified in a standard GST/TAP-pulldown, and experimenter bias in selecting proteins of interest is removed. The technique therefore compares very favorably with other commonly used techniques used in identification of novel protein-interactions (yeast 2-hybrid, GST/His or TAP Pulldowns).

## Disclosures

The authors declare that they have no competing financial interests.
